# The influence of phytase, pre-pellet cracked maize and dietary crude protein level on broiler performance via response surface methodology

**DOI:** 10.1186/s40104-019-0385-y

**Published:** 2019-10-04

**Authors:** Amy F. Moss, Peter V. Chrystal, Yueming Dersjant-Li, Peter H. Selle, Sonia Yun Liu

**Affiliations:** 1 Poultry Research Foundation within The University of Sydney, 425 Werombi Road, Camden, NSW 2570 Australia; 2 School of Environmental and Rural Science, University of New England, Armidale, NSW 2351 Australia; 3 School of Life and Environmental Sciences, Faculty of Science, The University of Sydney, Sydney, NSW 2006 Australia; 4 Baiada Poultry Pty Limited, Pendle Hill, NSW Australia; 5 Danisco Animal Nutrition, DuPont Industrial Biosciences, Marlborough, UK

**Keywords:** Crude protein, Maize, Phytase, Pre-pellet whole grain, Response surface

## Abstract

**Background:**

The reduction of crude protein levels in diets for broiler chickens may generate economic, environmental and flock welfare and health benefits; however, performance is usually compromised. Whole grain feeding and phytase may improve the utilization of reduced crude protein diets.

**Results:**

The effects of pre-pellet cracked maize (0, 15% and 30%) and phytase (0, 750 and 1500 FTU/kg) in iso-energetic maize-soy diets with three levels of crude protein (22%, 19.5% and 17%) were evaluated via a Box-Behnken response surface design. Each of 13 dietary treatments were offered to 6 replicate cages (6 birds/cage) of male Ross 308 broiler chicks from 7 to 28 d post-hatch. Model prediction and response surface plots were generated from experimental data via polynomial regression in R and only significant coefficients were included and discussed in the predicted models. Weight gain, feed intake and FCR were all influenced by pre-pellet cracked maize, phytase and crude protein level, where crude protein level had the greatest influence. Consequently, the reduction from 22% to 17% dietary crude protein in non-supplemented diets reduced weight gain, feed intake, relative gizzard weight, relative gizzard content and relative pancreas weight but improved FCR. However, the inclusion of 30% cracked maize to 17% crude protein diets restored gizzard weight and 1500 FTU phytase inclusion to 17% crude protein diets increased relative gizzard contents and pancreas weights. Cracked maize and phytase inclusion in tandem to 17% crude protein diets increased weight gain, feed intake and FCR; however, this FCR was still more efficient than broilers offered the non-supplemented 22% crude protein diet. Broilers offered the pre-pellet cracked maize and phytase inclusions reduced AME in 22% crude protein diets but improved AME by 2.92 MJ (14.16 versus 11.24 MJ; *P* < 0.001) in diets containing 17% crude protein. Ileal N digestibility was greater in broilers offered diets with 17% crude protein than those offered the 22% crude protein diet; irrespective of phytase and pre-pellet cracked maize.

**Conclusion:**

Pre-pellet cracked maize and phytase inclusions will improve the performance of broilers offered reduced crude protein diets.

## Background

There is a particular focus on the reduction of crude protein levels in diets for broiler chickens by replacing soybean meal with complementary amino acids as it has considerable potential to generate economic, environmental and flock welfare and health benefits [1]. Within the literature, amino acid inclusions are commonly termed ‘synthetic’ or ‘crystalline’; however, these terms are not strictly correct for all amino acids. Therefore, the term ‘complementary’ has been chosen in the present paper to describe these amino acids, as they are included to the diet to complement the protein-bound amino acids.

The successful implementation of reduced crude protein diets is problematic as bird performance is usually compromised by low dietary crude protein concentrations despite the inclusion of complementary amino acids to meet requirements. There are indications that reduced soybean meal and increased complementary amino acid inclusions in reduced crude protein diets reduce gizzard functionality [1] and may compromise bird performance. Therefore, whole grain feeding (WGF) should provide adequate stimulus to enhance gizzard weights, functionality and bird performance in the context of reduced crude protein diets.

While reduced crude protein diets axiomatically contain less phytate as inclusions of soybean meal are reduced, phytase has been reported to enhance amino acid digestibility and absorption. Phytase inclusions of 500 FTU/kg were reported to increase the digestibility coefficient of 16 amino acids by 49.7% (0.720 versus 0.481) in the proximal jejunum, 20.1% (0.801 versus 0.667) in the distal jejunum, 9.07% (0.878 versus 0.805) in the proximal ileum and 7.24% (0.904 versus 0.843) in the distal ileum in Truong et al. [2]. Additionally, the disappearance rate, or absorption, of the sum of 16 amino acids was increased by 58% (15.06 versus 23.77) in the proximal jejunum, 27% (20.91 versus 26.49) in the distal jejunum, 15% (25.33 versus 29.01) in the proximal ileum and 13% (26.49 versus 29.88) in the distal ileum. Thus, despite a reduction of dietary substrate, the extra-phosphoric effects of phytase enhance the digestibility of protein-bound amino acids by reducing protein-phytate complex formation [3] and may also improve amino acid absorption.

Furthermore, gizzard functionality may be related to phytase efficacy in broiler chickens [4]. Moss et al. [4] reported in whole barley diets, phytase supplementation reduced FCR by 3.2% whereas phytase supplementation increased FCR by 3.1% in ground barley diets. It is possible stronger gizzard function improves exogenous phytase efficacy because the gizzard is the main site of phytate degradation. Cracked or coarsely ground maize have been reported to improve nutrient utilization in broiler chicken [5]. Moreover, reduced protein diets typically contain less soybean meal and more complemental amino acids; therefore, it is more likely to contain less fiber and birds may generate smaller and less functional gizzard. Thus, the combination of phytase supplementation and cracked maize may work in tandem to alleviate reductions in performance associated with reducing dietary crude protein concentrations.

De Leon et al. [6] explored the possibility of using Box-Behnken response surface design (BBD) in poultry nutrition research, where multiple nutrients can be studied simultaneously with less number of treatments in comparison to full factorial design. For example, the present study included three factors and three levels of each factor, which requires 27 treatments by full factorial design; however, BBD only requires 13 treatments [7, 8]. Furthermore, response surface design visualizes the impact of each factor and compares the relative importance of any two factors [7, 8]. Therefore, the objective of the current study was to determine the effects of phytase (0, 750 and 1500 FTU/kg) and three levels of pre-pellet cracked maize inclusions (0, 15% and 30%) in maize-soy diets with three levels of crude protein (22%, 19.5% and 17%; basically, a ‘standard’ diet and two reduced crude protein/high complemental amino acid diets) via a Box Behnken response surface design. The hypotheses were reducing crude protein level would depress growth performance and feed efficiency; however, supplementation of phytase and inclusion of cracked maize would restore growth performance and nutrient utilizations in broiler chickens.

## Materials and methods

### Experimental design

A three factor, three level Box-Behnken design with 13 dietary treatments was chosen to determine the effects of three levels of phytase, cracked maize inclusion and crude protein level on the growth performance, gizzard characteristics, nutrient utilisation and protein (N) digestibility and disappearance rates of broilers. The three levels of each factor are shown in Table 1. Each of 13 dietary treatments were offered to six replicate cages (six birds per cage) or a total of 468 male Ross 308 broiler chicks over 7–28 d post-hatch. A common crumbled starter based on maize and soybean meal was offered to broiler chickens from 0 to 6 d post-hatch. The matrix of dietary treatments is shown in Table 2. Cracked maize were included in the diet prior to steam pelleting allow the determination of protein (N) digestibility without any discrepancies with dietary markers as those described in Moss et al. [9].

**Table 1:** Experimental factors and respective levels used in the Box-Behnken design

Factor	Level (− 1)	Level (0)	Level (+ 1)
Phytase, FTU/kg	0	750	1500
Crude protein, %	17	19.5	22
Cracked maize, %	0	15	30

**Table 2:** List of 13 experimental treatments of the Box-Behnken design offered to broiler chickens over 7–28 d post-hatch

Treatments	Phytase, FTU/kg	Crude protein, %	Cracked maize, %
1	0	17	15
2	0	19.5	0
3	0	19.5	30
4	0	22	15
5	750	17	0
6	750	17	30
7	750	19.5	15
8	750	22	0
9	750	22	30
10	1500	17	15
11	1500	19.5	0
12	1500	19.5	30
13	1500	22	15

### Diet preparation

A total of three iso-energetic maize-soy experimental diets were formulated to three crude protein levels; a standard 22% crude protein diet, and two reduced crude protein diets (19.5% and 17% crude protein) with increasing complementary amino acid inclusions (lysine, methionine, threonine, tryptophan, valine, arginine, isoleucine) to maintain a constant digestible lysine level. Ideal protein ratios for essential amino acids were kept constant where possible; however leucine, valine, arginine and isoleucine levels in the 22% crude protein diet exceeded formulated levels in reduced crude protein diets. Celite (Celite™ World Minerals, Lompoc, CA) was included in diets as an inert acid insoluble ash (AIA) marker to determine apparent digestibility coefficients of protein (N) in the distal ileum. Where appropriate, phytase (*Buttiauxella* sp. expressed in *Trichoderma reesei;* Axtra® PHY, Danisco Animal Nutrition, Marlborough, UK) was included to experimental diets over-the-top to nutrient adequate diets to the specified inclusion rate at the expense of Celite. A proportion of the maize was cracked and mixed into the diet prior to steam pelleting. The rest of maize was ground in a hammer mill through a 3.2-mm screen prior to incorporation to the diets. The nutrient composition of feed ingredients was analysed by NIR prior to formulation and the formulated diet composition and nutrient specifications of experimental diets are shown in Tables 3 and 4. Diets were pelleted through a cold pellet press at a temperature of 65 °C.

**Table 3:** Composition of dietary treatments

Items, g/kg	Diet 1	Diet 2	Diet 3	Diet 4	Diet 5	Diet 6	Diet 7	Diet 8	Diet 9	Diet 10	Diet 11	Diet 12	Diet 13
Maize ground^a^	545	598.8	298.8	368.4	695	395	448.8	518.4	218.4	545	598.8	298.8	368.4
Maize cracked^a^	150	0	300	150	0	300	150	0	300	150	0	300	150
Soybean meal^a^	216.9	308.5	308.5	382.6	216.9	216.9	308.5	382.6	382.6	216.9	308.5	308.5	382.6
Soybean oil	15.3	32.5	32.5	45.9	15.3	15.3	32.5	45.9	45.9	15.3	32.5	32.5	45.9
Lysine HCl	5.24	2.44	2.44	1.80	5.24	5.24	2.44	1.80	1.80	5.24	2.44	2.44	1.80
Methionine	3.98	3.18	3.18	2.54	3.98	3.98	3.18	2.54	2.54	3.98	3.18	3.18	2.54
Threonine	2.63	1.35	1.35	0.33	2.63	2.63	1.35	0.33	0.33	2.63	1.35	1.35	0.33
Tryptophan	0.31	0	0	0	0.31	0.31	0	0	0	0.31	0	0	0
Valine	2.71	1.13	1.13	0	2.71	2.71	1.13	0	0	2.71	1.13	1.13	0
Arginine	2.68	0.02	0.02	0	2.68	2.68	0.02	0	0	2.68	0.02	0.02	0
Isoleucine	2.28	0.69	0.69	0	2.28	2.28	0.69	0	0	2.28	0.69	0.69	0
Salt	0.53	1.43	1.43	2.15	0.53	0.53	1.43	2.15	2.15	0.53	1.43	1.43	2.15
Sodium bicarbonate	4.33	2.97	2.97	1.87	4.33	4.33	2.97	1.87	1.87	4.33	2.97	2.97	1.87
Limestone	10.70	10.58	10.58	10.47	10.70	10.70	10.58	10.47	10.47	10.70	10.58	10.58	10.47
Dicalcium phosphate	14.78	13.78	13.78	12.97	14.78	14.78	13.78	12.97	12.97	14.78	13.78	13.78	12.97
Choline chloride (60%)	0.6	0.6	0.6	0.6	0.6	0.6	0.6	0.6	0.6	0.6	0.6	0.6	0.6
Premix^b^	2.0	2.0	2.0	2.0	2.0	2.0	2.0	2.0	2.0	2.0	2.0	2.0	2.0
Phytase	0	0	0	0	0.075	0.075	0.075	0.075	0.075	0.15	0.15	0.15	0.15
Celite	20.0	20.0	20.0	20.0	19.9	19.9	19.9	19.9	19.9	19.9	19.9	19.9	19.9

^a^Feedstuffs were analysed via NIR prior to formulation; additionally, the maize and soybean meal were quantified to contain 5.5 mg/g and 13.82 mg/g of phytic acid, respectively

^b^The vitamin-mineral premix supplied per tonne of feed: retinol 12 MIU, cholecalciferol 5 MIU, tocopherol 50 g, menadione 3 g, thiamine 3 g, riboflavin 9 g, pyridoxine 5 g, cobalamin 0.025 g, niacin 50 g, pantothenate 18 g, folate 2 g, biotin 0.2 g, copper 20 g, iron 40 g, manganese 110 g, cobalt 0.25 g, iodine 1 g, molybdenum 2 g, zinc 90 g, selenium 0.3 g

**Table 4:** Formulated nutrient specifications of dietary treatments

Items, g/kg	Diet 1	Diet 2	Diet 3	Diet 4	Diet 5	Diet 6	Diet 7	Diet 8	Diet 9	Diet 10	Diet 11	Diet 12	Diet 13
AMEn, MJ/kg	12.60	12.60	12.60	12.60	12.60	12.60	12.60	12.60	12.60	12.60	12.60	12.60	12.60
Protein, %	170	195	195	220	170	170	195	220	220	170	195	195	220
Lysine^a^	11.20	11.20	11.20	11.20	11.20	11.20	11.20	11.20	11.20	11.20	11.20	11.20	11.20
Methionine^a^	6.10	5.72	5.72	5.41	6.10	6.10	5.72	5.41	5.41	6.10	5.72	5.72	5.41
Methionine + cysteine^a^	8.29	8.29	8.29	8.29	8.29	8.29	8.29	8.29	8.29	8.29	8.29	8.29	8.29
Threonine^a^	7.50	7.50	7.50	7.50	7.50	7.50	7.50	7.50	7.50	7.50	7.50	7.50	7.50
Tryptophan^a^	1.85	2.02	2.02	2.41	1.85	1.85	2.02	2.41	2.41	1.85	2.02	2.02	2.41
Isoleucine^a^	7.84	7.84	7.84	8.42	7.84	7.84	7.84	8.42	8.42	7.84	7.84	7.84	8.42
Leucine^a^	11.84	14.13	14.13	15.96	11.84	11.84	14.13	15.96	15.96	11.84	14.13	14.13	15.96
Arginine^a^	11.65	11.65	11.65	13.76	11.65	11.65	11.65	13.76	13.76	11.65	11.65	11.65	13.76
Valine^a^	8.96	8.96	8.96	9.09	8.96	8.96	8.96	9.09	9.09	8.96	8.96	8.96	9.09
Ca	8.00	8.00	8.00	8.00	8.00	8.00	8.00	8.00	8.00	8.00	8.00	8.00	8.00
Total phosphorus	6.01	6.21	6.21	6.37	6.01	6.01	6.21	6.37	6.37	6.01	6.21	6.21	6.37
Available P	4.00	4.00	4.00	4.00	4.00	4.00	4.00	4.00	4.00	4.00	4.00	4.00	4.00
Phytate-P	1.92	2.13	2.13	2.29	1.92	1.92	2.13	2.29	2.29	1.92	2.13	2.13	2.29
Chloride	2.20	2.20	2.20	2.20	2.20	2.20	2.20	2.20	2.20	2.20	2.20	2.20	2.20
Potassium	8.11	9.78	9.78	11.12	8.11	8.11	9.78	11.12	11.12	8.11	9.78	9.78	11.12
Sodium	1.60	1.60	1.60	1.60	1.60	1.60	1.60	1.60	1.60	1.60	1.60	1.60	1.60
Lipid	43.31	58.52	58.52	70.24	43.31	43.31	58.52	70.24	70.24	43.31	58.52	58.52	70.24
Fibre	20.36	21.56	21.56	22.48	20.36	20.36	21.56	22.48	22.48	20.36	21.56	21.56	22.48
Starch	458.8	396.2	396.2	343.8	458.8	458.8	396.2	343.8	343.8	458.8	396.2	396.2	343.8
Analysed phytase activity, FTU/kg	< 180	< 180	< 180	< 180	950	967	701	829	812	1255	1868	1632	968
Analysed protein (N × 6.25)	173.0	196.5	196.5	216.5	168.9	172.7	190.3	215.7	225.4	164.4	204.7	193.2	223.9

^a^Digestible basis

### Bird management

A total of 468 male Ross 308 chicks were obtained from a commercial hatchery and offered a proprietary starter diet from hatch to 7 d. At 7 d post-hatch birds were individually identified (wing-tags), weighed and allocated into bioassay cages based on body-weights so as to minimize variation within and between cages. Each of 13 dietary treatments was offered to six replicate cages (six birds per cage) during the 7 to 28 d post-hatch experimental period. Birds had unrestricted access to feed and water under a ‘16-h on, 8-h off’ lighting regime in an environmentally controlled facility. An initial room temperature of 32 ± 1 °C was maintained for the first week, which was gradually decreased to 22 ± 1 °C by the end of the third week. This study fully complied with specific guidelines approved by the Animal Ethics Committee of The University of Sydney (Project Number: 2016/973).

### Sample collection and chemical analysis

Initial and final body weights were determined and feed intakes recorded, from which feed conversion ratios (FCR) were calculated. Any dead or culled birds were removed daily and their body-weights recorded and used to adjust FCR calculations. Feed intakes, and excreta outputs were collected from 25 to 27 d post-hatch to calculate apparent metabolisable energy (AME), metabolisable to gross energy ratio (ME:GE ratio), nitrogen (N) retention and N-corrected AME (AMEn) on a dry matter basis. Over this total excreta collection period, water intakes were monitored to determine water to feed intake ratios. Excreta were dried for 24 h at 80 °C in an air-forced oven. The GE (gross energy) of diets and excreta were determined via bomb calorimetry using an adiabatic calorimeter (Parr 1281 bomb calorimeter, Parr Instruments Co., Moline, IL). AME (MJ/kg) was calculated by the following equation:

$$ {\mathrm{AME}}_{\mathrm{diet}}=\frac{\left(\mathrm{Feed}\ \mathrm{intake}\times {\mathrm{GE}}_{\mathrm{diet}}\right)-\left(\mathrm{Excreta}\ \mathrm{output}\times {\mathrm{GE}}_{\mathrm{excreta}}\right)}{\left(\mathrm{Feed}\ \mathrm{intake}\right)} $$

N-corrected AME values were calculated by correcting to zero N retention, using the factor of 36.54 kJ/g [10].

N retention was calculated by the following equation:

$$ \mathrm{Retention}\ \left(\%\right)=\frac{\left(\mathrm{Feed}\ \mathrm{intake}\times {\mathrm{Nutrient}}_{\mathrm{diet}}\right)-\left(\mathrm{Excreta}\ \mathrm{output}\times {\mathrm{Nutrient}}_{\mathrm{excreta}}\right)}{\left(\mathrm{Feed}\ \mathrm{intake}\times {\mathrm{Nutrient}}_{\mathrm{diet}}\right)}\times 100 $$

At d 28, birds were euthanised by an intravenous injection of sodium pentobarbitone. The pH of digesta within the gizzard was immediately determined in situ with an EZ Do model 7011 pH probe. Gizzard, gizzard contents and pancreas were then removed and weighed to determine their absolute and relative weights. The small intestine was removed, and the distal ileum was demarcated by the distal half of the point between Meckel’s diverticulum and the ileo-caecal junction. Digesta was collected in its entirety from the distal ileum by gently expressing each segment, pooling the samples by cage and the samples were then homogenised, freeze dried and weighed to determine the apparent digestibility of N. The N and AIA concentrations were determined as outlined in Siriwan et al. [11].

$$ \mathrm{DigestibilityCoefficient}=\frac{{\left(\mathrm{Nutrient}/\mathrm{AIA}\right)}_{\mathrm{diet}}-{\left(\mathrm{Nutrient}/\mathrm{AIA}\right)}_{\mathrm{digesta}}}{{\left(\mathrm{Nutrient}/\mathrm{AIA}\right)}_{\mathrm{diet}}} $$

Ileal protein disappearance rates were calculated from the following equation:

$$ \mathrm{Nutrient}{\mathrm{disappearancerate}}_{\left(\mathrm{g}/\mathrm{bird}/\mathrm{d}\right)}={\mathrm{Feedintake}}_{\left(\mathrm{g}/\mathrm{bird}\right)}\times {\mathrm{Dietarynutrient}}_{\left(\mathrm{g}/\mathrm{kg}\right)}\times {\mathrm{Nutrientdigestibility}}_{\left(\mathrm{apparentdigestibilitycoefficient}\right)} $$

Where FI is the 24 h feed intake immediately prior to euthanisation (g/bird), nutrient content_diet_ is the dietary protein (N) concentration (g/kg) and ADC is the apparent digestibility coefficients of protein (N).

Toe bone samples were collected from all birds by severing the middle toe through the joint between the 2^nd^ and 3^rd^ tarsal bones from the distal end. Toes from each cage were pooled and the composite samples dried to a constant weight at 100 °C and then ashed in a muffle furnace at 550 °C for 16 h for the assessment of bone mineralisation as described by Potter [12]. Distal ileal tissue samples were collected from one bird per cage, rinsed in RNA-free water and stored in RNAlater at − 80 °C for all replicate cages of treatments 2, 3, 6, 9, 11 and 12 for analysis of gene expression. For the gene expression analysis, total RNA isolation was carried out using TRIzol reagent (Invitrogen Life Technologies, Carlsbad, CA) according to the manufacturer’s instructions. The concentration and purity of total RNA measured by its optical density at 260 and 280 nm. One microgram of total RNA was reverse transcribed with a reverse transcription kit (Thermo Fisher) according to the manufacturer’s instructions. A quantitative real-time PCR assay was performed with the 7500 fluorescence detection system (Applied Biosystems, Foster City, CA) according to optimized PCR protocols using the SYBR-Green PCR kit (Thermo Fisher). Nested primers were designed (Table 5) within cloned chicken cDNA sequences with the Primer Express software. The PCR conditions were an initial denaturation step at 95 °C for 2 min, 40 cycles at 95 °C for 3 s and annealing and extension temperature of 60 °C for 30 s. To confirm amplification specificity, a melting curve analysis was performed on PCR products from each primer pair. Gene expression was quantified using the comparative threshold cycle method, and the data were expressed as the value relative to treatment 2 which contained the intermediary crude protein level and no phytase or whole grain inclusions.

**Table 5:** Primers used for quantitative real-time PCR

Genes	Accession number	Forward sequence (5′ to 3′)	Reverse sequence (5′ to 3′)	Role
*β-actin*	NM_205518	GAGAAATTGTGCGTGACATCA	CCTGAACCTCTCATTGCCA	Highly conserved, selected as the standard in the gene expression analysis
*GLUT-2*	Z22932	CCGCAGAAGGTGATAGAAGC	ATTGTCCCTGGAGGTGTT	Glucose transporter
*SGLT-1*	XM_415247	AGATTTGGAGGGCAGAGGAT	GCCCAAAGAGATTTGGATGA	Sodium dependant glucose transporter
*PepT-1*	AY029615	TACGCATACTGTCACCATCA	TCCTGAGAACGGACTGTAAT	Di- and tri-peptide transporter

### Statistical analysis

Model prediction, contour plots and response surface plots were generated from experimental data using polynomial regressions in R 3.3.3 with the package RSM. To generate models, non-significant coefficients were excluded, and the reduced equation recalculated for each response variable. The Akaike Information Criterion was used where more than one model was found to be significant. Model selection was conducted as demonstrated in Liu et al. [7], models chosen contained only significant parameters, had an insignificant lack of fit and the minimal number of parameters to produce the greatest multiple *R*^2^ value. The experimental units were replicate cage means and a probability level of less than 5% was considered to be statistically significant.

## Results

The average mortality rate during the experimental period was 2.35% and was not influenced by dietary treatment (*P* > 0.1). The influence of dietary treatments on growth performance, mortality rate, toe ash, gizzard characteristics and pancreas weight are shown in Table 6. The influence of dietary treatments on nutrient utilisation and protein (N) digestibility coefficients and disappearance rates are shown in Table 7. The influence of selected dietary treatments on relative mRNA expression are shown in Table 8.

**Table 6:** Treatment means for the 13 dietary treatments on growth performance and mortality rate from 7 to 28 d post-hatch and toe ash, relative gizzard weight, relative gizzard contents, relative pancreas weights and gizzard pH at 28 d post-hatch

Treatments	Weight gain, g/bird	Feed intake, g/bird	FCR, g/g	Mortality rate, %	Toe ash, %	Relative gizzard weight, g/kg	Relative gizzard contents, g/kg	Relative pancreas weight, g/kg	Gizzard pH
1	1239	1837	1.485	0.00	11.73	20.91	6.17	2.44	2.48
2	1109	1728	1.575	8.33	11.54	21.37	4.83	3.11	2.58
3	1169	1701	1.455	0.00	11.57	19.81	4.08	2.87	2.37
4	1135	1642	1.448	0.00	11.58	19.29	4.68	3.05	2.39
5	1212	1785	1.475	0.00	12.59	20.75	5.62	2.68	2.53
6	1210	1802	1.488	2.78	12.42	20.99	4.78	2.59	2.41
7	1194	1700	1.423	0.00	12.82	19.44	6.15	2.70	2.55
8	1130	1638	1.462	2.78	12.72	19.24	4.40	2.93	2.69
9	1132	1587	1.403	0.00	12.89	19.66	5.19	2.81	2.63
10	1208	1828	1.517	2.78	12.58	20.80	6.23	2.66	2.43
11	1250	1750	1.400	2.78	12.58	19.66	4.75	2.82	2.50
12	1185	1762	1.497	2.78	12.56	20.35	5.03	2.71	2.47
13	1104	1682	1.540	8.33	12.94	20.16	5.62	3.02	2.83
Mean	1175	1726	1.474	2.35	12.35	20.19	5.19	2.80	2.53
Standard Deviation	99	106	0.102	5.84	0.658	1.67	1.74	0.35	0.24

**Table 7:** Treatment means for the 13 dietary treatments on nutrient utilisation from 25 to 27 d post-hatch and ileal protein (N) digestibility coefficient and disappearance rates at 28 d post-hatch

Treatments	AME, MJ/kg DM	ME:GE, MJ/MJ	N retention, %	AMEn, MJ/kg DM	Protein (N) digestibility	Protein (N) disappearance rate, g/bird/d
1	13.18	0.817	21.10	12.24	0.856	20.74
2	12.98	0.803	23.05	12.03	0.822	21.25
3	13.03	0.801	22.92	12.00	0.822	20.94
4	13.04	0.780	23.67	12.08	0.812	22.00
5	13.13	0.826	20.48	12.18	0.826	18.95
6	13.23	0.836	21.86	12.11	0.841	19.94
7	13.01	0.805	22.41	12.07	0.821	20.23
8	12.91	0.780	23.98	11.95	0.783	21.11
9	13.13	0.792	25.64	12.08	0.810	22.08
10	12.97	0.824	20.14	12.07	0.822	18.82
11	12.95	0.794	24.09	11.88	0.834	22.77
12	13.04	0.804	22.85	12.06	0.835	21.66
13	13.17	0.793	26.01	12.17	0.841	24.12
Mean	13.06	0.804	22.94	12.07	0.825	21.12
Standard deviation	0.20	0.020	1.82	0.19	0.023	1.77

**Table 8:** Treatment means for selected dietary treatments on mRNA expression at 28 d post-hatch

Treatments	SGLT1	GLUT2	PEPT1
2	1.035	1.122	0.012
3	1.414	1.150	1.230
6	1.594	1.108	1.582
9	1.128	0.932	0.404
11	1.414	1.244	1.198
12	1.408	1.232	0.075
Mean	1.332	1.131	0.750
Standard deviation	0.778	0.559	1.026

Thus, the response of weight gain in broilers offered dietary treatments is described by the following equation (*R*^2^ = 0.991; *P* < 0.001):

$$ \mathrm{Gain}=0.832\times \mathrm{PHY}+59.005\times \mathrm{CP}+40.279\times \mathrm{CM}-0.042\times \mathrm{PHY}\times \mathrm{CP}-2.051\times \mathrm{CP}\times \mathrm{CM} $$

**Fig. 1:**
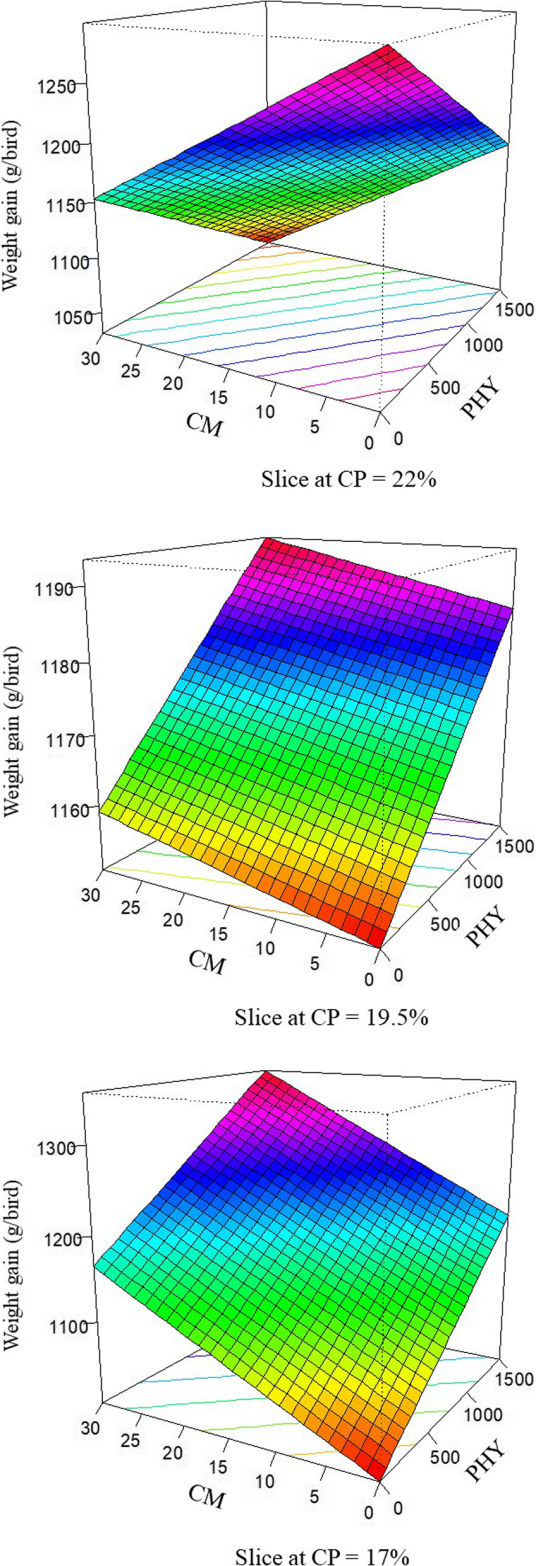
Response surface plot displaying the effect of phytase and pre-pellet cracked maize on weight gain, g/bird, over three levels of crude protein from 7 to 28 d post-hatch in broiler chickens

Where PHY represents the phytase inclusion, CP represents the level of crude protein and CM represents the pre-pellet cracked maize inclusion. The response surface plots for weight gain modelled from the above equation are demonstrated in Fig. 1. At the standard 22% crude protein diet, the pre-pellet cracked maize and phytase inclusions depressed the weight gain of broiler chickens. However, when crude protein level was decreased to 19.5% and 17% with increased complemental amino acid inclusion, 30% cracked maize and 1500 FTU/kg phytase generated the greatest weight gain.

The response of feed intake in broilers offered dietary treatments is described by the following equation (*R*^2^ = 0.994; *P* < 0.001):

$$ \mathrm{Intake}=1.153\times \mathrm{PHY}+87.080\times \mathrm{CP}+71.483\times \mathrm{CM}-0.058\times \mathrm{PHY}\times \mathrm{CP}-3.660\times \mathrm{CP}\times \mathrm{CM} $$

**Fig. 2:**
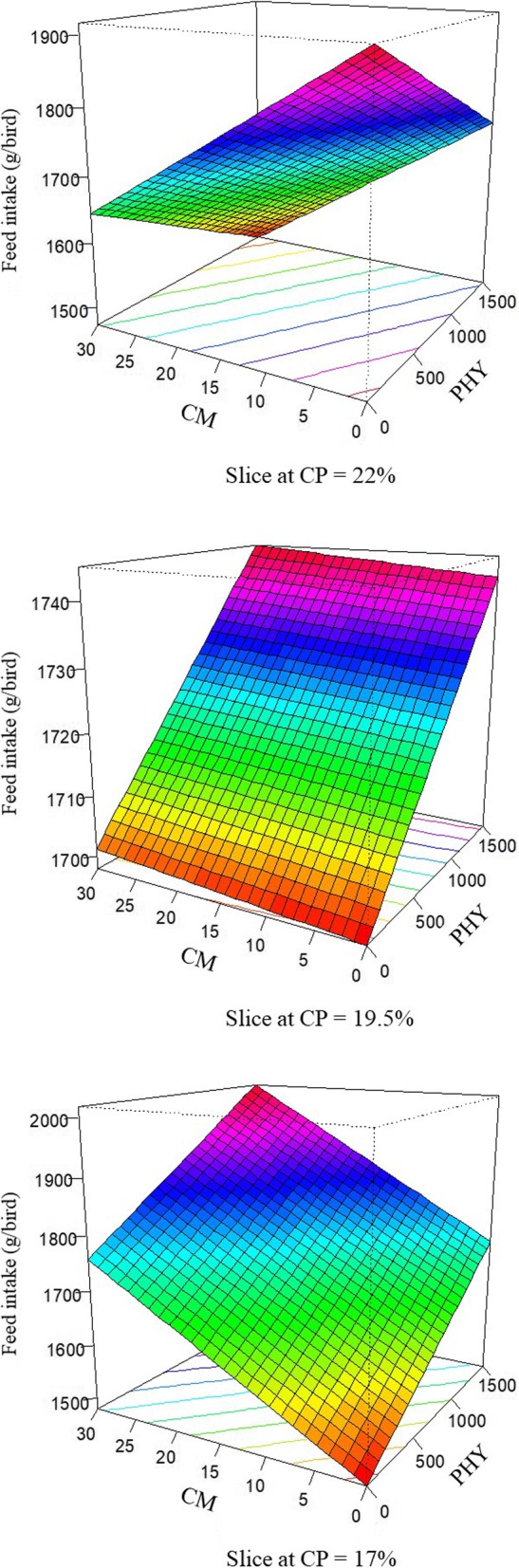
Response surface plot displaying the effect of phytase and pre-pellet cracked maize on feed intake, g/bird, over three levels of crude protein from 7 to 28 d post-hatch in broiler chickens

The response surface plots for feed intake modelled from the above equation are demonstrated in Fig. 2. Similar to weight gain, at the standard 22% crude protein diet, the pre-pellet cracked maize and phytase inclusions depressed feed intake in broiler chickens. However, when crude protein level was decreased to 19.5% and 17% with complemental amino acid inclusion, 30% cracked maize and 1500 FTU/kg phytase generated the greatest intake.

The response of FCR in broilers offered dietary treatments is described by the following equation (*R*^2^ = 0.993; *P* < 0.001):

$$ \mathrm{FCR}=6.796\times {10}^{-4}\mathrm{PHY}+7.543\times {10}^{-2}\mathrm{CP}+5.061\times {10}^{-2}\mathrm{CM}-3.459\times {10}^{-5}\mathrm{PHY}\times \mathrm{CP}-2.607\times {10}^{-3}\mathrm{CP}\times \mathrm{CM} $$

**Fig. 3:**
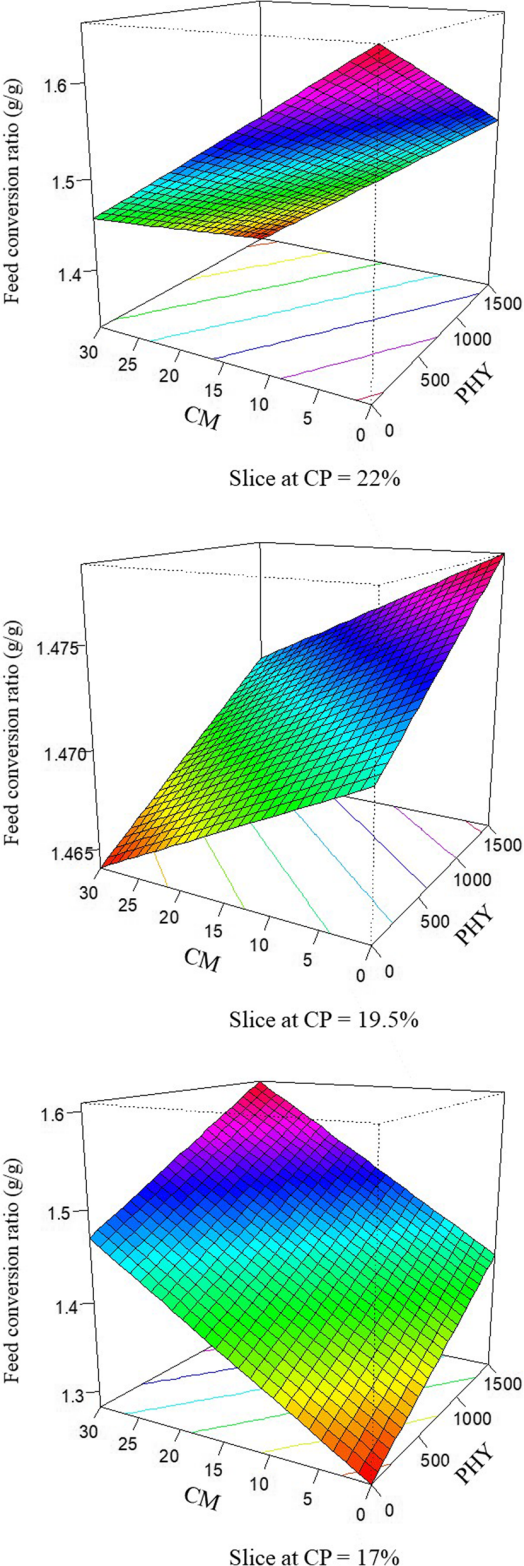
Response surface plot displaying the effect of phytase and pre-pellet cracked maize on feed conversion ratio, FCR, g/g, over three levels of crude protein from 7 to 28 d post-hatch in broiler chickens

The response surface plots for FCR modelled from the above equation are demonstrated in Fig. 3. At the standard 22% crude protein diet, broilers offered the 30% pre-pellet cracked maize and 1500 FTU/kg phytase inclusions generated the most efficient feed conversion. However, pre-pellet cracked maize and phytase inclusion in tandem to 17% CP diets depressed FCR.

The response of relative gizzard weights (g/kg) in broilers offered dietary treatments is described by the following equation (*R*^2^ = 0.987; *P* < 0.001):

$$ \mathrm{Relative}\ \mathrm{gizzard}\ \mathrm{weight}=1.025\times \mathrm{CP}+1.108\times \mathrm{CM}-0.056\times \mathrm{CP}\times \mathrm{CM} $$

Diets with 22% CP, 0% CM possessed a gizzard size of 22.6 g/kg. As the crude protein level decreased to 17%, relative gizzard weight also decreased to 17.4 g/kg. However, the addition of 30% CM to 17% CP diets restored relative gizzard weight to 22.1 g/kg.

The response of relative pancreas weight (g/kg) in broilers offered dietary treatments is described by the following equation (*R*^2^ = 0.987; *P* < 0.001):

$$ \mathrm{Relative}\ \mathrm{pancreas}\ \mathrm{weight}=1.442\times {10}^{-3}\times \mathrm{PHY}+1.446\times {10}^{-1}\times \mathrm{CP}-7.566\times {10}^{-5}\times \mathrm{PHY}\times \mathrm{CP} $$

Similarly, reducing crude protein level from 22% crude protein to 17% crude protein reduced relative pancreas weights from approximately 3.1 g/kg to 2.6 g/kg. Phytase inclusion to reduced crude protein diets had little effect; however, phytase inclusion to standard 22% crude protein diets reduced relative pancreas weights.

The response of gizzard pH in broilers offered dietary treatments is described by the following equation (*R*^2^ = 0.990; *P* < 0.001):

$$ \mathrm{Gizzard}\ \mathrm{pH}=0.131\times \mathrm{CP}+0.079\times \mathrm{CM}-0.004\times \mathrm{CP}\times \mathrm{CM} $$

Reducing crude protein level from 22% crude protein to 17% crude protein reduced gizzard pH from 2.8 to 2.35. Pre-pellet cracked maize inclusion to reduced crude protein diets had little effect; however, cracked maize reduced gizzard pH in the standard 22% crude protein diets.

The response of percentage toe ash in broilers offered dietary treatments is described by the following equation (*R*^2^ = 0.995; *P* < 0.001):

$$ \mathrm{Toe}\ \mathrm{ash}=6.155\times {10}^{-3}\times \mathrm{PHY}+6.004\times {10}^{-1}\times \mathrm{CP}+2.940\times {10}^{-1}\times \mathrm{CM}-2.765\times {10}^{-4}\times \mathrm{PHY}\times \mathrm{CP}-1.493\times {10}^{-2}\times \mathrm{CP}\times \mathrm{CM} $$

**Fig. 4:**
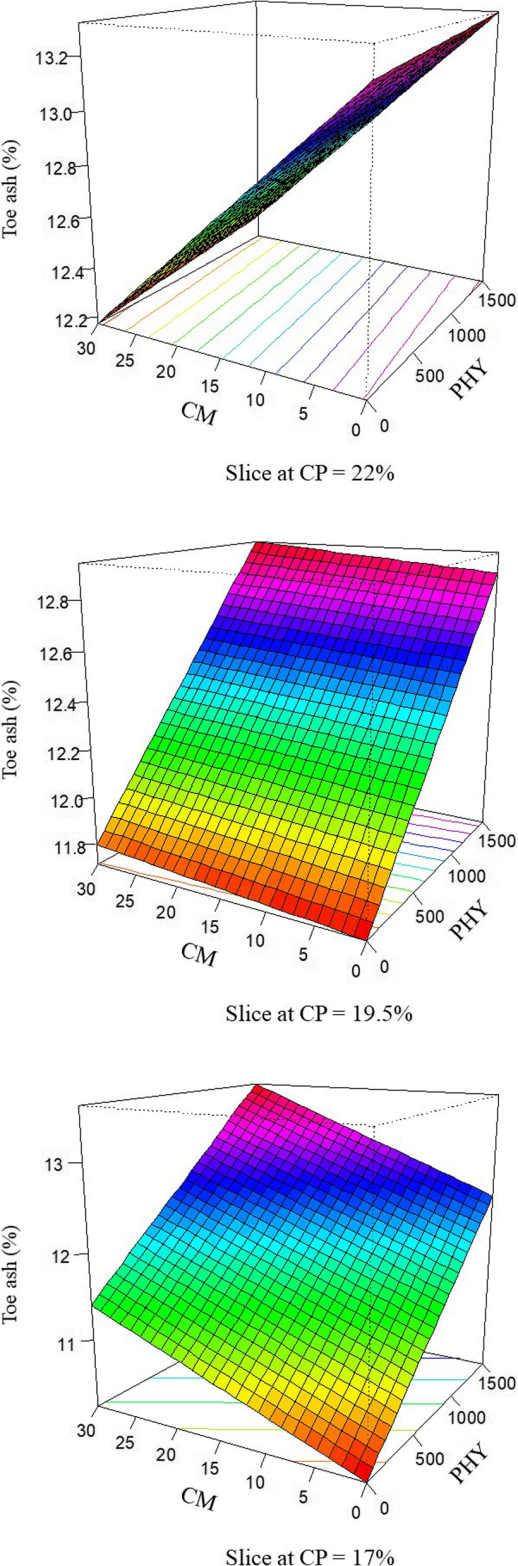
Response surface plot displaying the effect of phytase and pre-pellet cracked maize on toe ash, % over three levels of crude protein from at 28 d post-hatch in broiler chickens

The response surface plots for percentage toe ash modelled from the above equation are shown in Fig. 4. Reducing crude protein level from 22% crude protein to 17% crude protein reduced percentage toe ash. Within the 22% crude protein diet phytase tended to increase toe ash and pre-pellet cracked maize inclusions reduced toe ash. However, in the 19.5% and 17% reduced crude protein diets phytase inclusion had the largest influence and phytase and pre-pellet cracked maize inclusions in tandem increased percentage toe ash to 13.59%.

The response of AME (MJ/kg DM) in broilers offered dietary treatments is described by the following equation (*R*^2^ = 0.996; *P* < 0.001):

$$ \mathrm{AME}=6.648\times {10}^{-3}\times \mathrm{PHY}+6.613\times {10}^{-1}\times \mathrm{CP}+3.656\times {10}^{-1}\times \mathrm{CM}-3.387\times {10}^{-4}\times \mathrm{PHY}\times \mathrm{CP}-1.840\times {10}^{-2}\times \mathrm{CP}\times \mathrm{CM} $$

**Fig. 5:**
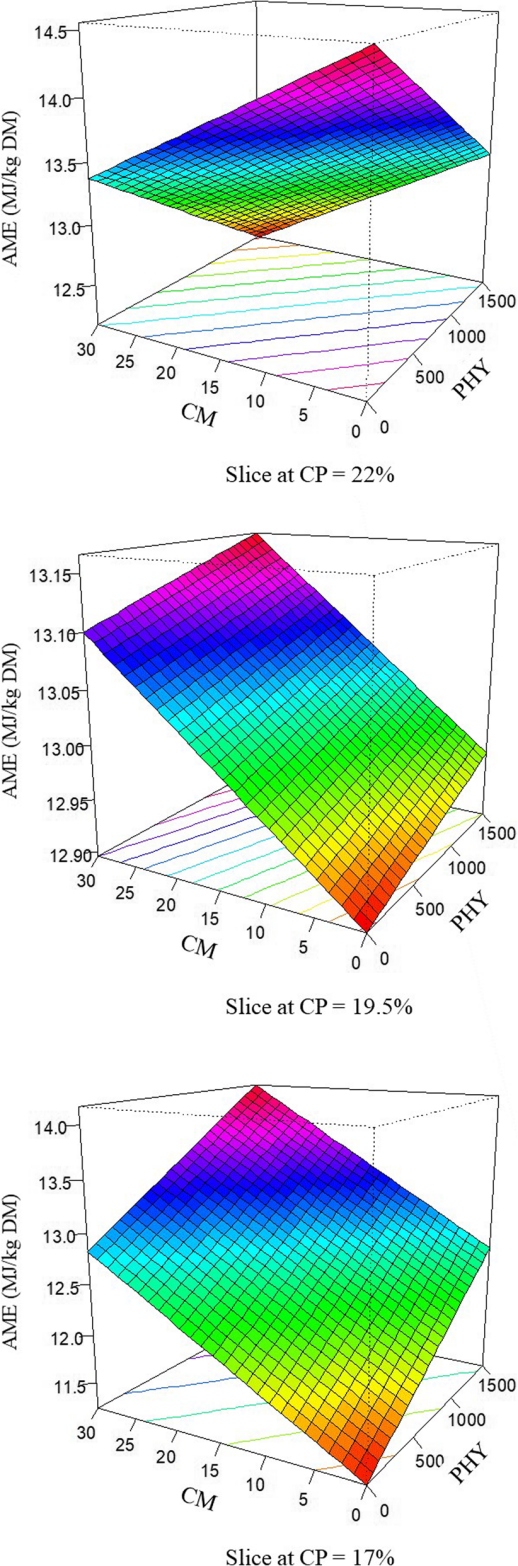
Response surface plot displaying the effect of phytase and pre-pellet cracked maize on AME, MJ/kg DM over three levels of crude protein from 25 to 27 d post-hatch in broiler chickens

The response surface plots for AME modelled from the above equation are demonstrated in Fig. 5. Pre-pellet cracked maize and phytase inclusion in tandem to 17% CP diets increased AME from 12.17 MJ in diets containing 22% crude protein to 14.16 MJ in diets containing 17% crude protein.

The response of N retention in broilers offered dietary treatments is described by the following equation (*R*^2^ = 0.998; *P* < 0.001):

$$ \mathrm{N}\ \mathrm{retention}\ \left(\%\right)=4.0393\times {10}^{-3}\times \mathrm{PHY}+1.155\times \mathrm{CP}-1.82\times {10}^{-4}\times \mathrm{PHY}\times \mathrm{CP} $$

**Fig. 6:**
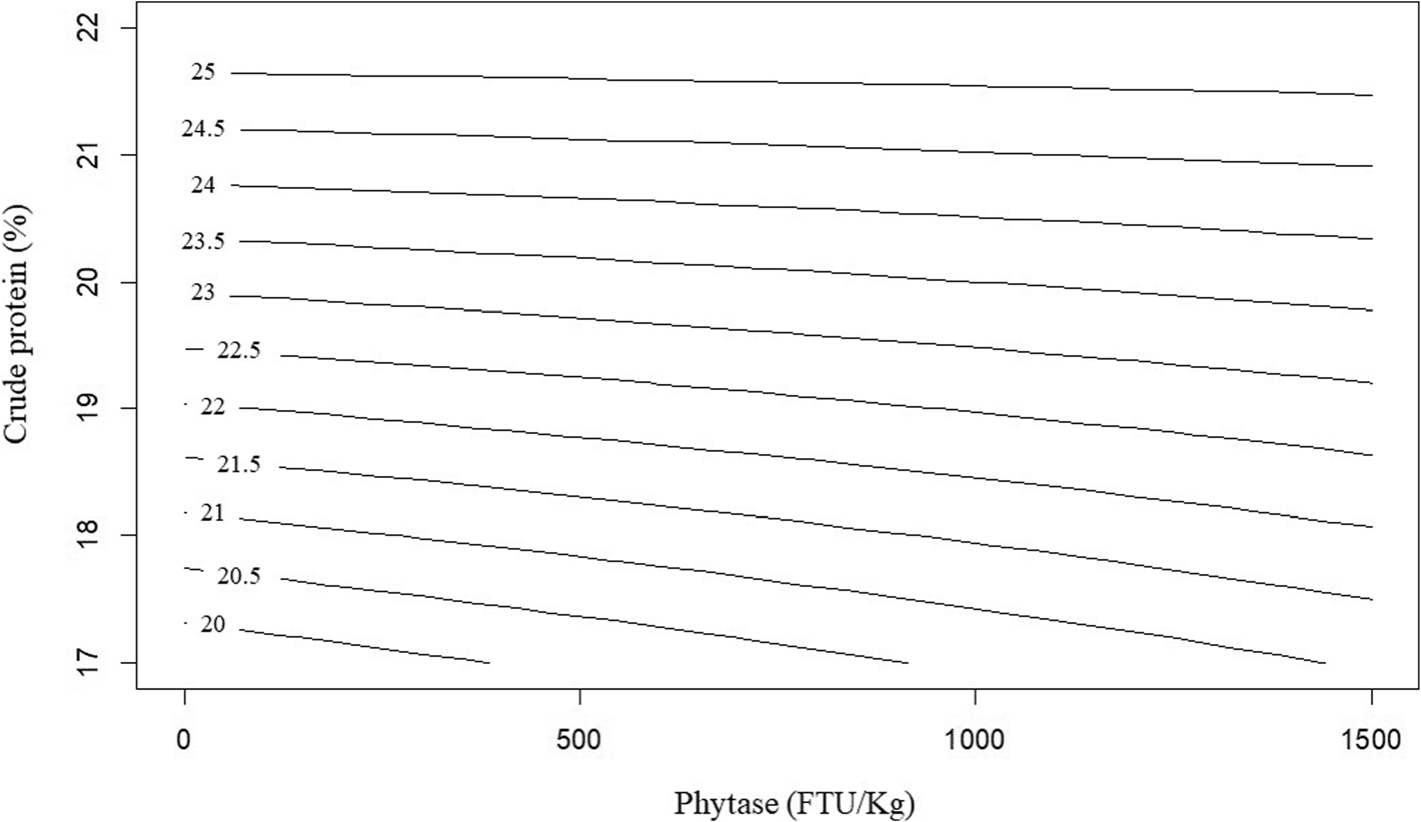
Contour plot displaying the effect of crude protein level, % and phytase inclusion, FTU/kg on N retention (%) over 25 to 27 d post-hatch in broiler chickens

The contour plot for N retention modelled from the above equation is demonstrated in Fig. 6. Reducing crude protein level from 22% to 17% reduced N retention from 25.41% to 19.64% in diets without phytase supplementation. Phytase inclusion increased N retention in diets with reduced crude proteinfrom 19.64% to 21.05% with 1500 FTU/kg phytase inclusion to diets with 17% crude protein. There was no significant response of AMEn in broilers offered dietary treatments.

The apparent protein (N) digestibility coefficients and disappearance rates in distal ileum were only influenced by dietary crude protein levels. The response of ileal protein digestibility coefficients in broilers offered dietary treatments is described by the following linear relationship (*R*^2^ = 0.417; *P* < 0.001):

$$ \mathrm{Ileal}\ \mathrm{N}\ \mathrm{digestibility}\ \mathrm{coefficient}=0.922-0.005\times \mathrm{CP} $$

Additionally, the response of ileal protein disappearance rates in broilers offered dietary treatments is described by the following equation (*R*^2^ = 0.605; *P* < 0.001):

$$ \mathrm{Ileal}\ \mathrm{N}\ \mathrm{disappearance}\ \mathrm{rate}\left(\mathrm{g}/\mathrm{bird}/\mathrm{d}\right)=10.541+0.543\times \mathrm{CP} $$

Reducing crude protein level from 22% crude protein to 17% crude protein increased ileal N digestibility, however, N disappearance rates were depressed with the transition to 17% crude protein diets.

The effects of phytase and cracked maize inclusion to diets with 19.5% crude protein on mRNA expression of SGLT1, GLUT2 and PEPT1 at the ileum of broilers are shown in Table 9. There was a significant treatment interaction (*P* < 0.005) where inclusion of cracked maize increased PEPT1 mRNA expression in the ileum of broilers offered diets without phytase; however, cracked maize inclusion significantly depressed PEPT1 mRNA expression in the ileum of broilers offered diets supplemented with exogenous phytase. There was no influence of phytase or cracked maize inclusions on SGLT1 or GLUT2 mRNA expression. The effects of two crude protein levels (to diets with 750 FTU/kg phytase and 15% cracked maize) on mRNA expression of SGLT1, GLUT2 and PEPT1 at 28 d post-hatch are also shown in Table 9. Broilers offered diets containing the 17% crude protein diet tended (*P* < 0.08) to express greater amounts of PEPT1 in the ileum than broilers offered diets containing 22% crude protein. There was no influence of crude protein level on SGLT1 or GLUT2 relative mRNA expression.

**Table 9:** The effects of phytase and whole grain (to diets with 19.5% crude protein) on mRNA expression of SGLT1, GLUT2 and PEPT1 and the effects of two crude protein levels (to diets with 750 FTU/kg phytase and 15% whole grain) on mRNA expression of SGLT1, GLUT2 and PEPT1 at 28 d post-hatch

Treatments		SGLT1	GLUT2	PEPT1
Phytase, FTU/kg	Whole grain, %			
0	0	1.035	1.122	0.012
0	30	1.414	1.150	1.230
1500	0	1.414	1.244	1.198
1500	30	1.408	1.232	0.075
SEM		0.191	0.228	0.323
Main effects				
Phytase				
0		1.225	1.136	0.621
1500		1.411	1.238	0.636
Whole grain				
0		1.224	1.183	0.605
30		1.411	1.191	0.652
Significance (*P* = )				
Phytase		0.341	0.660	0.962
Whole grain		0.340	0.972	0.885
Phytase × whole grain		0.326	0.929	0.002
Crude protein level, %				
17		1.594	1.108	1.582
22		1.278	0.932	0.404
SEM		0.268	0.268	0.426
LSD		-	-	-
Significance (*P* = )		0.652	0.652	0.079

## Discussion

In the present study, the reduction from 22% to 17% dietary crude protein in non-supplemented diets reduced weight gain from 1298 to 1003 g/bird and feed intake from 1915 to 1480 g/bird. Previously, reducing dietary crude protein from 23% to 17% significantly compromised weight gain and feed intake in Namroud et al. [13]. Phytase and pre-pellet cracked maize inclusions in tandem improved weight gain by 34% (1343 versus 1003 g/bird) and feed intake by 36% (2009 versus 1480 g/bird) in broilers offered diets with 17% crude protein. However, in diets with 1500 FTU/kg phytase and 30% pre-pellet cracked maize, broiler chickens offered diets containing 17% crude protein had worse feed conversion efficiency than birds offered diets with 22% crude protein. Linear models were found to have the best fit when predicting growth performance; however, the response of performance parameters to phytase and pre-pellet cracked maize inclusion is expected to plateau in accordance with the law of diminishing returns. Thus, a broader range of inclusion levels may need to be investigated to capture the optimal inclusion of phytase and pre-pellet cracked maize.

Reduced crude protein diets axiomatically contain less phytate and less intact protein due to the reduced inclusion of soybean meal and increased level of complemental amino acids (Table 4). Nevertheless, responses in weight gain and toe ash were still observed in broilers offered diets with 17% crude protein. The phytase to phytate ratio was effectively increased in reduced crude protein diets. In reduced crude protein diets, essential amino acids are formulated to meet broiler requirements; however, non-essential amino acids not included in the formulation may become a limiting factor. For example, previous studies [14, 15] reported the potential influence of glycine and serine on growth performance in reduced protein diets and suggested a minimal requirement for glycine and serine needs to be considered when formulating reduced protein diets. Unfortunately, in the present study, the requirement of glycine was not considered which might have contributed to the depression of growth performance in diets containing 17% CP. Supplementation of exogenous phytase has shown to improve the digestibility of both essential and non-essential amino acids [2]; hence, phytase inclusion may ultimately benefit broiler performance in reduced crude protein diets. It was unexpected that the 1500 FTU phytase inclusion did not improve weight gain in the standard 22% crude protein diet. Ideally, both mineral and amino acid matrixes should apply to phytase supplementation when formulating broiler diets; however, it is recommended to use different matrix values for different phytase inclusion rate. In order to avoid this confounding factor, matrix values were not assigned in the present study. The 22% crude protein diets contained inclusions of amino acids above requirements; including leucine, isoleucine, arginine and valine. Thus, this may explain the lack of response to phytase supplementation in diets containing standard levels of crude protein (22%).

Phytase and pre-pellet cracked maize in tandem generated substantial improvements in weight gain and feed intake in the present study. Phytase and whole grain inclusions have been previously reported to generate pronounced performance improvements when offered in tandem [4, 16]. The enhancement of exogenous phytase efficacy by larger particle size or whole grain inclusion is likely due to greater gizzard functionality. Whole grain feeding generates heavier and presumably more functional gizzards [17]. This is of importance as the gizzard is the prime site of phytate degradation by exogenous phytase [18]. The degradation of phytate is pivotal in the prevention of de novo binary protein-phytate complex formation. Thus, it follows that improved gizzard functionality generated by whole grain or larger particle size will advantage exogenous phytase efficacy.

Reduced crude protein diets may depress gizzard functionality, likely due to a reduction of dietary fibre [1]. Thus, the inclusion of cracked maize was expected to generate a pronounced response in gizzard weight and enhance the efficacy of phytase inclusions to reduced crude protein diets. In the present study, 22% crude protein diets without pre-pellet cracked maize inclusion possessed a typical gizzard weight of 22 g/kg [17]. As crude protein levels decreased from 22% to 17%, dietary fibre was reduced from 22.48 g/kg to 20.36 g/kg (Table 4). Moreover, particle size in reduced CP diets may be reduced because of higher inclusion of complemental amino acids. Therefore, the reduction in fibre and particle size may have contributed to the decline of relative gizzard weight in diets containing 17% CP(17.4 g/kg). Predictably, pre-pellet cracked maize inclusion to diets with 17% crude protein increased gizzard weight by 27% (22.1 versus 17.4 g/kg), effectively restoring gizzard weight to equal that of the 22% crude protein diet. In contrast, inclusions of pre-pellet cracked maize to the 22% CP diets slightly reduced gizzard weight. The diameter of pelletizer screen is 4.0 mm in our feed mill; therefore, it is most likely particle size of cracked maize was further reduced during conditioning and pelleting. Thus, pre-pellet inclusion of cracked maize did not duplicate the benefit in standard CP diets as reported in the literature.

Reducing crude protein level from 22% crude protein to 17% crude protein lowered relative pancreas weights from approximately 3.1 g/kg to 2.6 g/kg and gizzard pH from 2.8 to 2.35. Reduced crude protein diets have been previously reported to reduce pancreas weights [1] and may reflect the replacement of intact protein which requires digestion with complementary amino acids. The reduction of gizzard pH in reduced crude protein diets is beneficial to improve digestibility but will also advantage phytase efficacy by creating a more acidic environment increasing phytate solubility [19].

In the present study, reducing dietary crude protein from 22% to 17% reduced AME and ME:GE ratios. In the reduction of dietary crude protein, maize inclusions increased from 518.4 to 695 g/kg in the transition from 22% to 17% crude protein and consequently, lipid content decreased from 70.24 g/kg to 43.31 g/kg. This effectively creates a shift in energy dynamic towards energy derived from starch, with approximately 50% of dietary energy provided by starch in 22% crude protein diets shifting to 65% of dietary energy provided by starch in 17% crude protein diets. The increasing dependency on glucose for energy might have overwhelmed glucose transport systems and was previously proposed to increase competition between amino acids and glucose for sodium-dependent transport [1].

Phytase has been shown to manipulate the digestive dynamics of protein as 1000 FTU/kg phytase significantly increased the average ileal digestibility of 17 amino acids by 12.3% (0.840 versus 0.748) in Amerah et al. [20] and 500 FTU/kg phytase increased the average digestibility of 16 amino acids by 49.7% (0.720 versus 0.481) in the proximal jejunum, 20.1% (0.801 versus 0.667) in the distal jejunum, 9.07% (0.878 versus 0.805) in the proximal ileum and 7.24% (0.904 versus 0.843) in the distal ileum in Truong et al. [2], effectively creating a proximal shift in amino acid digestibility. In the present study phytase was not found to significantly influence ileal protein digestibility coefficients; however, 1500 FTU/kg phytase improved N retention from 19.64% to 21.05% in broilers offered 17% crude protein diets. Ileal N digestibility was greater in broilers offered diets with 17% crude protein than those offered the 22% crude protein diet. Furthermore, reduce crude protein diets also tended (*P* < 0.08) to express more PEPT1 in the ileum, which should upregulate sodium-independent transport of di- and tri-peptides and likely improved ileal protein digestibility. Interestingly, there was a significant treatment interaction (*P* < 0.005) where inclusion of whole grain increased PEPT1 mRNA expression in the ileum of broilers offered diets without phytase and significantly depressed PEPT1 mRNA expression in the ileum of broilers offered diets supplemented with exogenous phytase. The mechanism behind this interaction is unclear; however, it is an indication that phytase and whole grain feeding in tandem are manipulating protein digestive dynamics.

## Conclusion

The indications are that decreasing dietary crude protein level depressed weight gain and feed conversion; however, pre-pellet cracked maize and phytase inclusions will improve the performance of broilers offered reduced crude protein diets. In the present study, the impact of cracked maize on gizzard function and protein digestibility was confounded by secondary particle size reduction during steam pelleting. Further studies on the effect of larger particle size and non-essential amino acids on growth performance and nutrient utilizations are required to explore the possibilities of successfully developing recued protein diets.

## Data Availability

Data may be provided following request to the corresponding author.

## Abbreviations

AIA: Acid insoluble ash
AME: Apparent metabolisable energy
AMEn: N corrected apparent metabolisable energy
CM: Cracked maize
CP: Crude protein
FTU: Phytase units
GE: Gross energy
ME:GE ratio: Metabolisable energy to gross energy
N: Nitrogen
NIR: Near-infrared spectroscopy
